# Midazolam impedes lung carcinoma cell proliferation and migration via EGFR/MEK/ERK signaling pathway

**DOI:** 10.1515/med-2023-0730

**Published:** 2023-06-05

**Authors:** Xiangchao Zhang, Zhe Han, Zhengjun Li, Tao Wang

**Affiliations:** Department of Anesthesiology, Shengyang Chest Hospital, Shenyang City, Liaoning 110044, China; Department of Anesthesiology, General Hospital of Northern Theater Command, Shenyang City, Liaoning 110015, China; Department of Thoracic Surgery, Shengyang Chest Hospital, Shenyang City, Liaoning 110044, China; Department of Anesthesiology, Shengyang Chest Hospital, No. 11 Beihai Street, Dadong District, Shenyang City, Liaoning 110044, China

**Keywords:** midazolam, non-small cell lung cancer, EGFR, MEK/ERK

## Abstract

Non-small-cell lung cancer (NSCLC) is a dominating type of lung cancer with high morbidity and mortality. Midazolam has been reported to promote cell apoptosis in NSCLC, but the molecular mechanism of midazolam remains to be further explored. In the current work, cell viability, proliferation, migration, and apoptosis rates of NSCLC cells treated with midazolam were measured using cell counting kit-8 assay, 5-ethynyl-2′-deoxyuridine (EdU) and colony formation assays, transwell, and flow cytometry assay, respectively, to evaluate the malignant behaviors. Western blot was applied to access EGFR/MEK/ERK pathway-related protein levels. The results demonstrated midazolam significantly declined the viability of NSCLC cells. Furthermore, midazolam restrained cell proliferation and migration and contributed to cell apoptosis in NSCLC. Midazolam exerted suppressive function to EGFR pathway during NSCLC development. Moreover, the activation of EGFR/MEK/ERK pathway abrogated the effects of midazolam on NSCLC cell proliferation, apoptosis, and migration. Taken together, midazolam exhibited anti-tumor effects hallmarked by EGFR pathway inhibition, providing a novel insight into the treatment of NSCLC.

## 1 Introduction

Lung cancer is one of the leading malignant tumors responsible for cancer-related mortality, accounting for about 26% of all deaths ascribed to cancer around the world [1,2]. The epidemiological report predicts approximately 2.1 million new cases and 1.8 million patients succumbed to lung cancer annually [3]. As a main category of lung cancer, non-small-cell lung cancer (NSCLC) constitutes more than 80% of all lung carcinomas [4]. The overall 5-year survival of NSCLC remains far from satisfactory due to the difficulty in early diagnosis and high postoperative recurrence rate [5]. Accordingly, it is imperative to expound the potential pathogenesis of NSCLC and find novel therapeutic strategies.

Surgical excision is the preferred option for solid tumors, including lung cancer [6]. It is reported that operative pain, intubation, extubation, and other surgical procedures can provoke serious complications by causing acute stress responses, influencing the operative effect as well as the prognosis and rehabilitation of patients [7]. Mounting evidence has confirmed that anesthetic agents execute inhibitory functions in the tumorigenesis and progression of human cancer [8,9]. Midazolam is a member of the benzodiazepine family of anesthetics and has antagonistic effects on anxiety epilepsy and muscle spasms and possesses hypnotic and sedative properties [10]. Midazolam is one of the most commonly used anesthetic agents during lung cancer surgery [11]. As reported by previous studies, the role of midazolam in regulating cell behaviors in lung cancer has been explored. Sun et al. [12] demonstrated that midazolam reduced cisplatin resistance in cisplatin-resistant NSCLC cells by regulating the miR-194-5p/HOOK3 axis, implying that midazolam could be used as an adjuvant drug for NSCLC treatment in clinical practices. Additionally, Zhang et al. [7] found that midazolam treatment inhibited the inflammatory responses in the patients of thoracoscopic resection of lung cancer. Thus, exploring the potential mechanism of midazolam in the development of NSCLC is of great significance.

Epidermal growth factor (EGF) is a critical regulator in numerous biological processes, including cell differentiation, stemness, proliferation, metastasis, and apoptosis [13]. Epidermal growth factor receptor (EGFR) is a tyrosine kinase receptor and plays a crucial role in the initiation and developemnt of multiple malignancies, such as lung cancer, breast cancer, glioblastoma, and colon cancer [14]. Manipulation of EGFR signaling has been proven practical to target cancer of multiple subtypes [15,16,17]. Recently, more and more studies focus on the role of EGFR/MEK/ERK pathways in lung cancer. For example, Chen et al. [18] found that chaetoglobosin G obviously inhibited A549 cell proliferation by inducing autophagy of A549 cells through EGFR/MEK/ERK pathway. Bae et al. [19] confirmed that the EGFR/MEK/ERK signaling would be a promising molecular target to control abnormal invasion in the epithelial–mesenchymal transition-induced NSCLC. Additionally, it has been reported that MEK/ERK signaling is a vital downstream pathway of EGFR and participates in the carcinogenesis and development of NSCLC [20]. Nevertheless, there are no investigations regarding the association between midazolam and EGFR/MEK/ERK signaling pathway so far.

In the current study, we aimed to provide new insights into the underlying mechanism of midazolam in the cell biology of NSCLC. Our results demonstrated that midazolam exhibited anti-tumor effects on NSCLC by regulating EGFR/MEK/ERK pathway.

## 2 Methods

### 2.1 Cell culture and treatment

Two human NSCLC cell lines SK-MES-1 and A549 were supplied by American Type Culture Collection (ATCC, USA) and maintained in dulbecco’s modified eagle medium (DMEM) (Invitrogen, USA) supplemented with 10% fetal bovine serum (FBS) (ThermoFisher, USA) in the presence of 5% CO_2_ at 37°C. To assess the role of midazolam in NSCLC, SK-MES-1 and A549 cells were treated with a series of different doses of midazolam (25, 50, 100, 200, and 400 μM) purchased from Sigma-Aldrich for 24 h. To activate the EGFR/MEK/ERK signaling pathway, the cells were treated with recombinant EGF (20 ng/ml, PeproTech, USA) for 48 h following midazolam treatment.

### 2.2 Cell Counting Kit-8 (CCK-8) assay

The viability of SK-MES-1 and A549 cells was detected by CCK-8 (Beyotime, Shanghai, China) according to the product manuals. NSCLC cells were seeded to 96-well plates at 2  ×  10^3^ cells per well and treated with midazolam, and then cultured at 37°C for 24 h. Afterward, each well was added with 10 μl CCK-8 solution. Two hours of incubation later, the optical density value was measured at 450 nm with a microplate reader (BioTek Instruments, USA).

### 2.3 5-Ethynyl-2′-deoxyuridine (EdU) assay

The proliferative capability of SK-MES-1 and A549 cells was measured by EdU labeling kit obtained from RiboBio (Guangzhou, China) complying with the manufacturer’s directions. SK-MES-1 and A549 cells were inoculated into 96-well plates at a density of 1 × 10^6^ cells/ml and treated with midazolam for 24 h. Afterward, 50 μM EdU was added to treat cells for 2 h, followed by fixation in 4% paraformaldehyde and thereafter stained by Apollo. Cell nuclear staining was performed with DAPI. The stained cells were observed under a fluorescence microscopy (Nikon, Japan) and counted with ImageJ software.

### 2.4 Colony formation assay

Cell proliferation was assessed with a colony formation assay. Briefly, 500 SK-MES-1 and A549 cells were plated to each well of six-well plates, treated with midazolam for 24 h, and cultivated at 37°C for about 14 days. The fresh medium was replaced every 2–3 days. Then, SK-MES-1 and A549 cells were immobilized with 4% paraformaldehyde, followed by dyed using 1% crystal violet and photographed by a microscope (Olympus, Japan). The number of colonies over 50 cells was counted.

### 2.5 Cell apoptosis assay

The apoptosis of SK-MES-1 and A549 cells was tested by the FITC Annexin V apoptosis detection kit (BD Biosciences, USA) in accordance with the instructions after treatment with midazolam for 18 h. SK-MES-1 and A549 cells were trypsinized, washed twice using PBS, and centrifugated for 10 min at 3,000 × *g*. Then, cells were harvested and resuspended in 1× binding buffer, counterstained with AnnexinV-FITC and PI, followed by incubation away from light for 10 min and analyzed with the FACScalibur flow cytometer (Becton Dickinson, USA).

### 2.6 Transwell migration assay

Transwell assay was conducted for the estimation of SK-MES-1 and A549 cell migration via using transwell chambers with 8-μm pores (Corning Inc., USA). Following corresponding treatments, SK-MES-1 and A549 cells resuspending in 100 μl serum-free medium were added to the upper transwell chambers. Meanwhile, the bottom of chambers was supplemented with 500 μl DMEM containing 20% FBS. At 24 h after incubation, cells on the surface of upper chambers were removed and then migrated cells were fixed in 4% paraformaldehyde, dyed with 0.1% crystal violet, and visualized by a microscope. Images captured from five random view fields were adopted to evaluate cell migratory ability. As for midazolam and EGF treatment, the midazolam or EGF mixed solution was added in the upper chamber for subsequent experiments.

### 2.7 Western blot analysis

Total protein extraction was performed with RIPA lysis buffer (Beyotime). The BCA protein detection kit (Beyotime) was applied for protein quantification. Equal amounts of protein samples were detached on 10% SDS-PAGE, transferred onto PVDF membranes (Millipore, USA), and blocked in 5% defatted milk. PVDF membranes were subjected to overnight incubation with primary antibodies (all from Abacam, USA) for EGFR (ab52894, 1/1,000), phosphorylated EGFR (p-EGFR, ab40815, 1/500), MEK (ab32091, 1/1,000), phosphorylated MEK (p-MEK, ab96379, 1/1,000), ERK (ab32537, 1/1,000), phosphorylated ERK (p-ERK, ab131438, 1/500), and GAPDH (ab8245, 1/500) at 4°C and subsequently probed with HRP-labeled secondary antibodies (ab6721, 1/2,000) for 1.5 h at room temperature and detected on an enhanced chemiluminescence test system (Thermo Fisher Scientific, USA). GAPDH served as an internal reference.

### 2.8 Xenograft model

0.2 ml of A549 cell suspension (1.0 × 10^7^/ml) was inoculated subcutaneously in the axilla of BALB/c nude mice aged 5–6 weeks to build a xenograft model of lung cancer. BALB/c nude mice were from the animal center of Shengyang Chest Hospital. After inoculation, the changes in transplanted tumor were observed closely. When the transplanted tumor was larger than 5.0 mm on day 7, it was determined as tumor formation. Six nude mice were randomly divided into the midazolam group and the control group, with three mice in each group. The midazolam group was injected with 0.5 ml (0.8 mg/kg) midazolam in the tumors, once a day at the same time for 28 consecutive days; the control group was injected with 0.5 ml normal saline, and the injection site was the same as midazolam group. The tumor volume and autonomous behavior of nude mice were recorded every 7 days. The diameter of the tumors was measured with a ruler and volumes (in mm^3^) were calculated using the formula (length × width^2^)/2. After 28 days, the nude mice were sacrificed, and the transplanted tumors were removed and weighed. The animal study was approved by the Institutional Animal Care and Use Committee (IACUC) of Shengyang Chest Hospital.

### 2.9 Statistical analysis

Statistical analysis was carried out using SPSS 20.0 software (IBM, USA). All results were represented as mean ± SD and each assay was conducted in triplicate. Comparison between the two groups was estimated by Student’s *t*-test. Differences among multiple groups were analyzed with one-way ANOVA. Statistical significance was deemed when *P*  <  0.05.

## 3 Results

### 3.1 Midazolam suppressed the viability of SK-MES-1 and A549 cells

**Figure 1:**
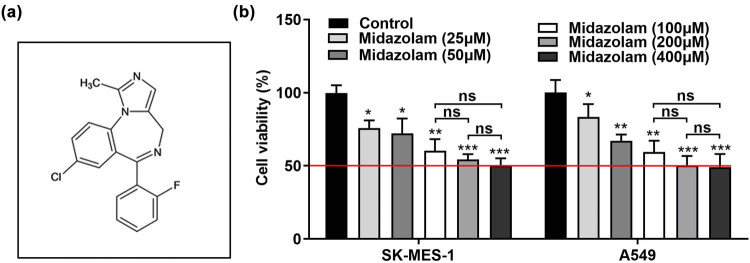
Midazolam suppressed the viability of SK-MES-1 and A549 cells. (a) The chemical structure of midazolam. (b) CCK-8 assay was performed to examine the viability of SK-MES-1 and A549 cells treated with different concentrations of midazolam (25, 50, 100, 200, and 400 μM). All results were represented as mean ± SD and each assay was conducted in triplicate. ^*^ *P*  <  0.05, ^**^ *P*  <  0.01^, ***^ *P*  <  0.001.

The structure of midazolam is featured with benzodiazepine pharmacological activities. As shown in Figure 1a, the chemical structure of midazolam was characterized by the 5-fluorophenyl-1,4-benzodiazepine ring. To certify the characteristic of midazolam in NSCLC progression, SK-MES-1 and A549 cells were treated with increasing concentrations of midazolam for 24 h. Subsequently, the effects of midazolam on cell viability were evaluated by the CCK-8 assay. We observed that the viability of SK-MES-1 and A549 cells was gradually descended by midazolam, and midazolam at 100, 200, and 400 μM inhibited SK-MES-1 and A549 cells’ viability by nearly 50% (Figure 1b). Based on the above findings, we concluded that midazolam led to the inhibition of NSCLC cell viability.

### 3.2 Midazolam retarded cell proliferation and migration and promoted cell apoptosis in SK-MES-1 and A549 cells

**Figure 2:**
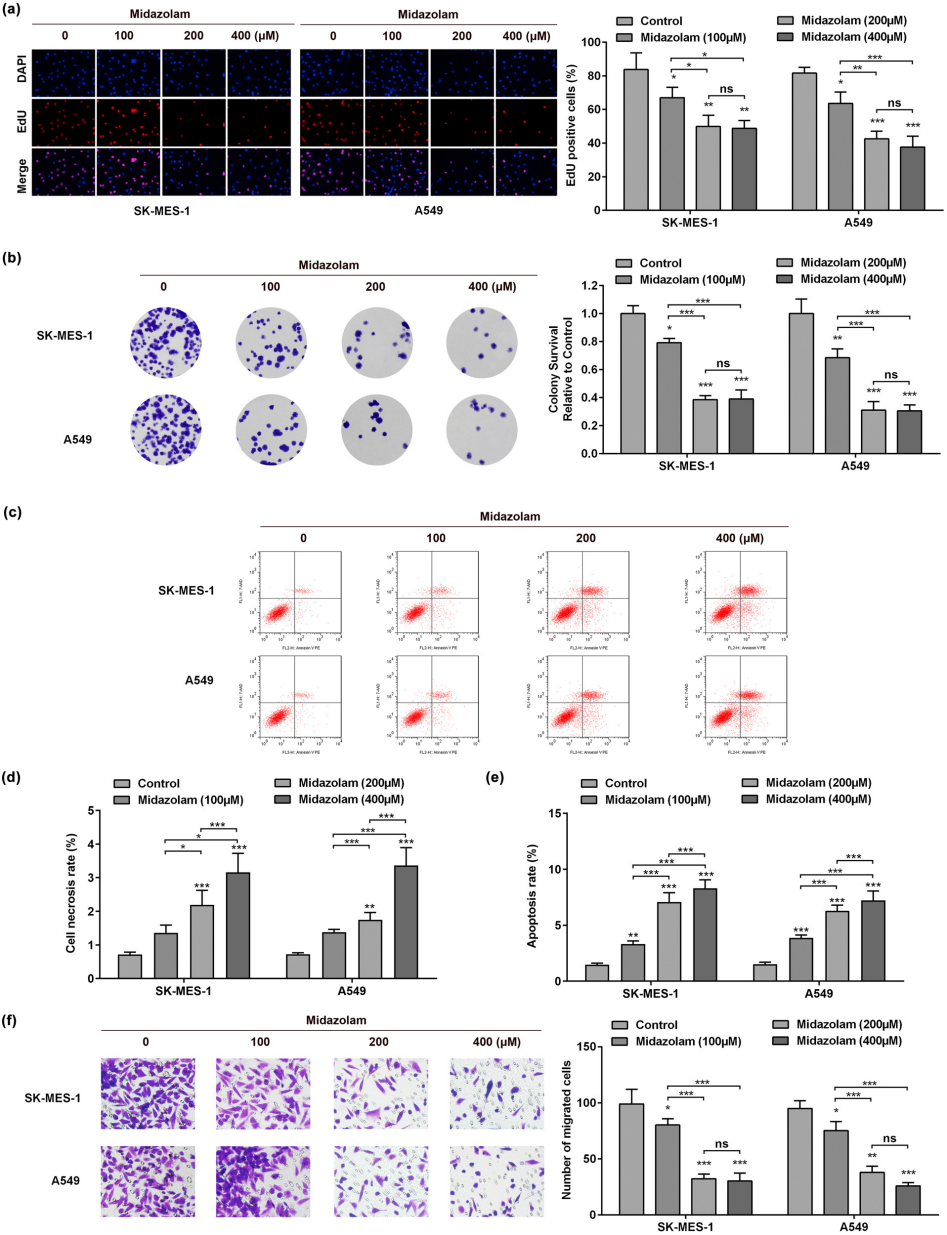
Midazolam retarded cell proliferation and migration and promoted cell apoptosis in SK-MES-1 and A549 cells. (a) After SK-MES-1 and A549 cells were treated with 100, 200, or 400 μM midazolam, cell proliferation was detected by EdU staining assay. (b) Colony formation assay was carried out to determine the colony-forming capacity of SK-MES-1 and A549 cells exposed to 100, 200, or 400 μM midazolam. (c) Flow cytometry scatter diagram under the midazolam treatment. The role of midazolam in the (d) necrosis and (e) early apoptosis of SK-MES-1 and A549 cells was estimated by flow cytometry analysis. (f) Transwell assay was adopted to evaluate the influences of midazolam on cell migration in NSCLC. All results were represented as mean ± SD and each assay was conducted in triplicate. ^*^ *P*  <  0.05, ^**^ *P*  <  0.01, ^***^ *P*  <  0.001.

Given that midazolam overtly repressed the viability of SK-MES-1 and A549 cells at the concentrations of 100, 200, and 400 μM, NSCLC cells were treated with these three doses of midazolam for subsequent use. The EdU assay showed that midazolam obviously decreases the number of EdU-positive cells (Figure 2a). Moreover, midazolam suppressed the proliferative capacity of SK-MES-1 and A549 cells (Figure 2b). Consistently, midazolam promoted necrosis and early apoptosis of SK-MES-1 and A549 cells (Figure 2c–e). Furthermore, the migration ability of NSCLC cell was significantly decreased by midazolam (Figure 2f).

### 3.3 Midazolam hindered the activation of EGFR/MEK/ERK signaling pathway

**Figure 3:**
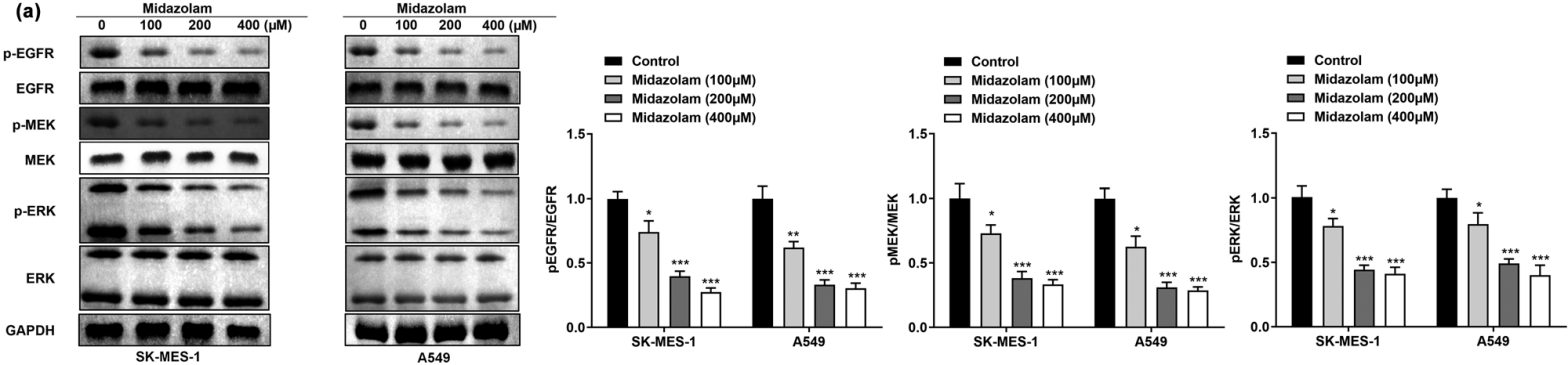
Midazolam hindered the activation of EGFR/MEK/ERK signaling pathway. (a) Following SK-MES-1 and A549 cells underwent different midazolam treatments, western blot assay was performed to detect the activation of EGFR, MEK, and ERK in NSCLC. All results were represented as mean ± SD and each assay was conducted in triplicate. ^*^ *P*  <  0.05, ^**^ *P*  <  0.01, ^***^ *P*  <  0.001.

EGFR/MEK/ERK signaling pathway plays an essential role in the initiation and progression of NSCLC. We, therefore, explored the association between midazolam and EGFR/MEK/ERK pathway. As displayed in Figure 3a, midazolam significantly declined the phosphorylation level of EGFR. Hence, the phosphorylation of downstream MEK and ERK in SK-MES-1 and A549 cells was significantly repressed by midazolam treatment. Taken together, these data illustrated that midazolam suppressed the activation of EGFR-mediated MEK/ERK signaling pathway. Considering that both 200 and 400 μM midazolam can significantly inhibit the growth of SK-MES-1 and A549 cells and there is no statistical difference between two groups, 200 μM was selected for subsequent experiments.

### 3.4 EGFR/MEK/ERK signaling pathway was participated in midazolam-inhibited the malignant behaviors of NSCLC cells

**Figure 4:**
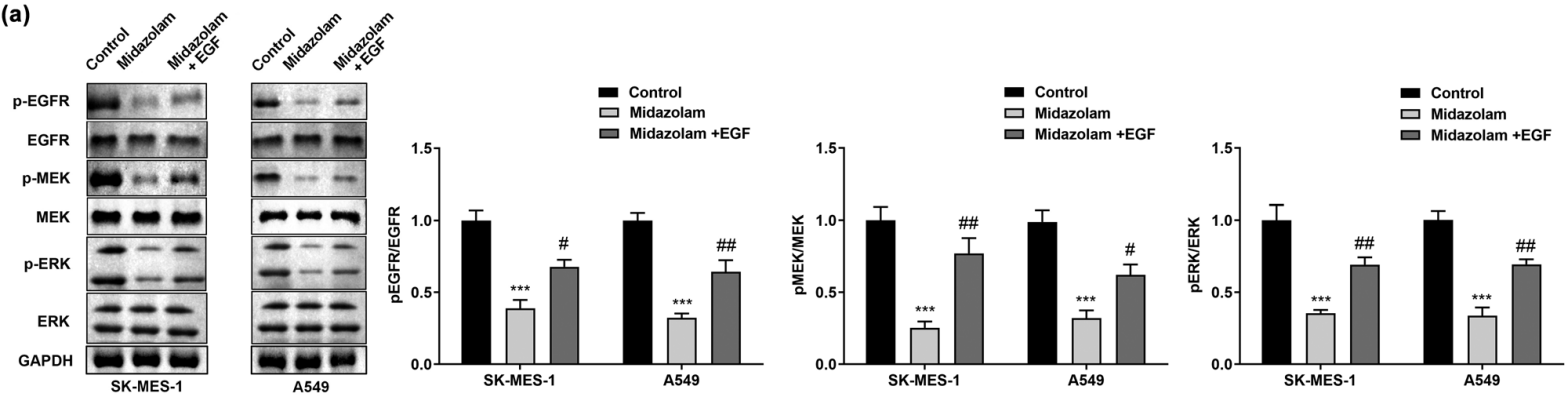
The regulatory effects of midazolam on MEK/ERK signaling pathway were mediated by inhibiting EGFR phosphorylation. (a) After SK-MES-1 and A549 cells were exposed to midazolam for 48 h, western blot was implemented to measure the activation of EGFR, MEK and ERK. All results were represented as mean ± SD and each assay was conducted in triplicate. ^***^ *P*  <  0.001, compared with the control group. ^#^ *P*  <  0.001, ^##^ *P*  <  0.001, compared with the midazolam group.

**Figure 5:**
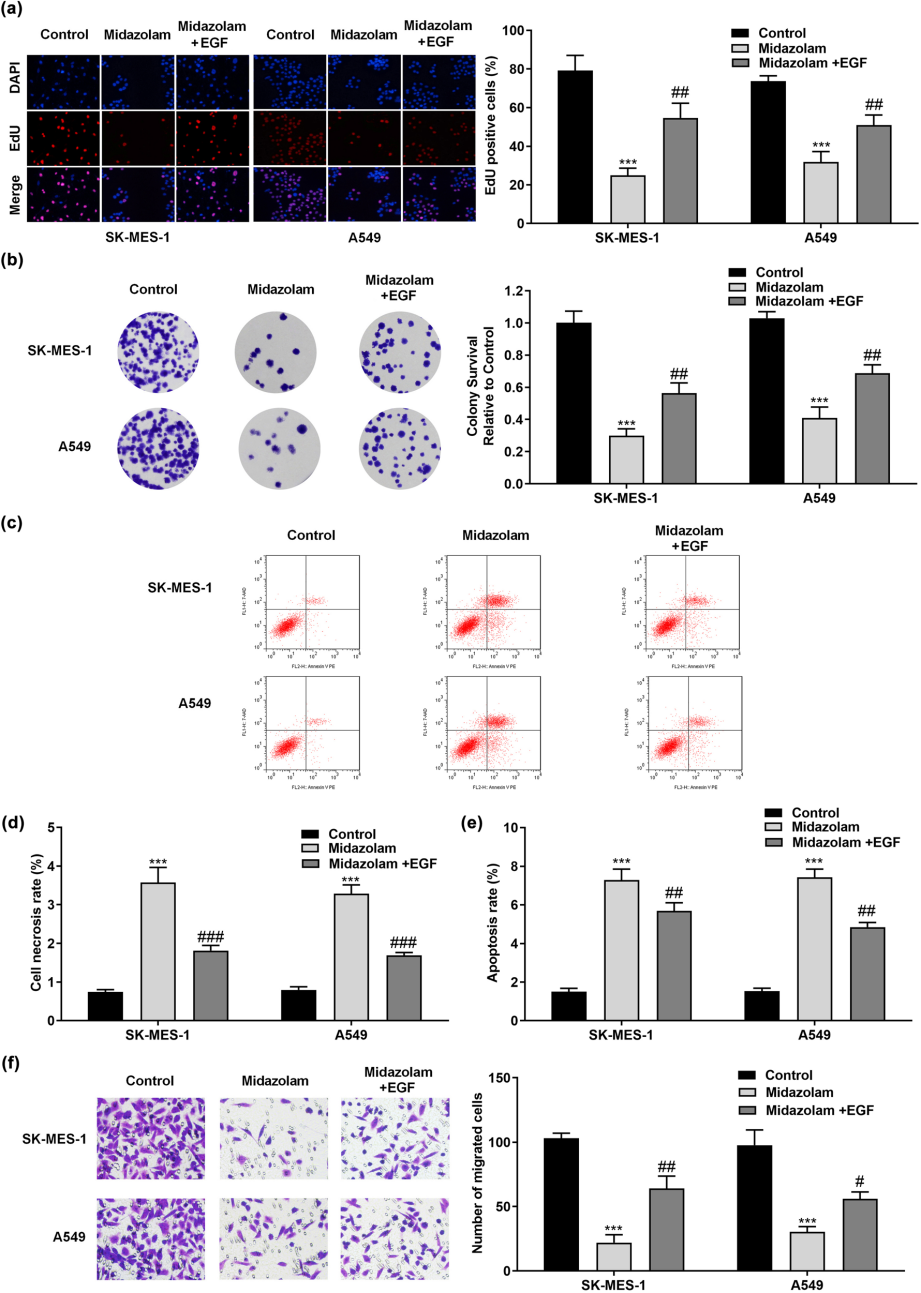
EGFR/MEK/ERK signaling pathway participated in midazolam-mediated cell carcinogenesis in NSCLC. (a) Following different treatments, an EdU staining assay was conducted for the estimation of NSCLC cell proliferation. (b) The proliferative ability of SK-MES-1 and A549 cells under corresponding treatments was also determined by colony formation assay. (c) Flow cytometry scatter diagram of different groups. The (d) necrosis and (e) early apoptosis of SK-MES-1 and A549 cells in different groups was detected with flow cytometry analysis. (f) Transwell assay was carried out to assess the migration of NSCLC cells from different groups. All results were represented as mean ± SD and each assay was conducted in triplicate. ^***^ *P*  <  0.001, compared with the control group. ^#^ *P*  <  0.001, ^##^ *P*  <  0.001, compared with the midazolam group.

To validate the suppression of EGFR pathway postmidazolam treatment, we conducted a rescue experiment by EGF stimulation. As shown in Figure 4, recombinant EGF partially offset the midazolam-induced reduction in the phosphorylation of vital factors EGFR, MEK, and ERK in EGFR/MEK/ERK signaling pathway, which suggested that recombinant EGF treatment gave rise to the activation of EGFR-dependent MEK/ERK pathway. The proliferative ability of SK-MES-1 and A549 cells weakened by midazolam treatment was partially offset when EGFR/MEK/ERK signaling pathway was activated (Figure 5a). Similarly, the results of the colony formation assay manifested that the number of colonies decreased by administration of midazolam was partially offset by the activation of EGFR/MEK/ERK pathway (Figure 5b). In addition, midazolam-induced necrosis and apoptosis of NSCLC cells were partially offset by EGF treatment (Figure 5c–e). In agreement with the above findings, we observed that activation of EGFR-mediated MEK/ERK pathway partially offset the repressive role of midazolam in NSCLC cell migration (Figure 5f).

### 3.5 Midazolam suppressed cell growth *in vivo*

**Figure 6:**
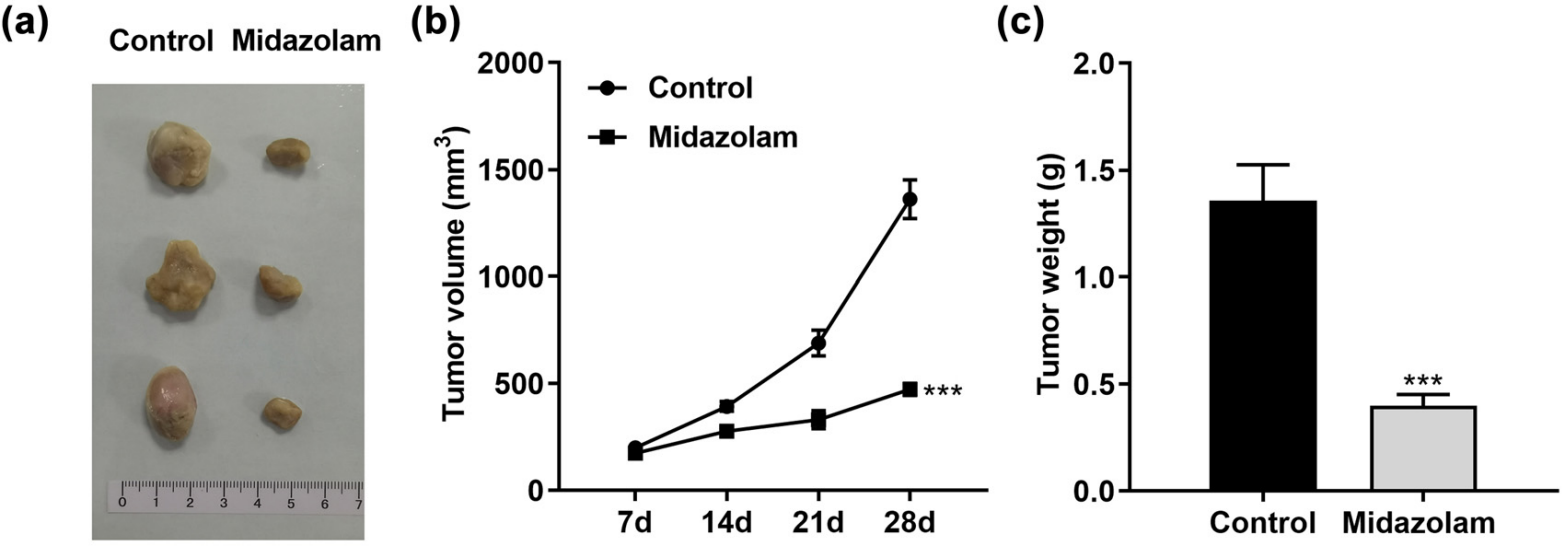
Midazolam suppressed cell growth *in vivo*. (a) Images, (b) tumor volume, and (c) tumor weight of nude-mouse transplanted tumor model. ^***^ *P*  <  0.001.

To explore of the inhibitory effect of midazolam on cancer growth is applicable *in vivo*, we performed Xenograft Model. The mouse Autonomous Behavior Evaluation form (Table A1) suggested that midazolam (0.8 mg/kg) has no significant effect on the autonomous behavior of mice. As indicated in Figure 6a, the tumor diameter of the control group was about 1.0 cm, while that of the group treated with midazolam was significantly reduced to about 0.5 cm. Furthermore, tumor volume (Figure 6b) and tumor weight (Figure 6c) were both prominently downregulated by midazolam treatment compared with the control group. Taken together, our data suggested that midazolam suppressed NSCLC cells’ growth and development *in vivo*.

## 4 Discussion

Lung cancer is one of the most common malignancies with striking morbidity and mortality across the globe, resulting in a crushing burden on public health [21]. NSCLC is a predominant pathological type of lung cancer, with a 5-year survival rate of only 18% [22,23]. Notably, a large proportion of patients with NSCLC are usually in the advanced stage and exhibit tumor metastasis once diagnosed [24,25]. In spite of tremendous advance in the development of early diagnosis and prevention, the prognosis of NSCLC patients is still frustrating [26]. Therefore, it is urgent to find new methods that can treat NSCLC and explore the specific mechanism in the process of NSCLC.

Midazolam is an extensively applied anesthetic in cancer surgery and presents significant pharmacological effects on sedation, anti-anxiety, anti-convulsion, hypnosis, and muscle relaxation [7]. Multiple lines of evidence illustrate that midazolam plays a vital role in a wide range of cancers via affecting cell activities, including cell proliferation, apoptosis, migration, and invasion [27,28,29]. More importantly, Jiao et al. revealed that midazolam performed anti-tumor properties in the progression of lung cancer [30]. In this study, midazolam repressed cell proliferation and migration and facilitated cell apoptosis in NSCLC. The inhibitory effects of midazolam on the malignant behaviors of NSCLC cells exerted its anti-tumor function in NSCLC, which was consistent with a previous study [12]. Interstingly, a compound with a similar structure of midazolam named 3-indolyl cyclopent[b]indoles is known to inhibit EGFR pathway in NSCLC cells and colon cancer cells [31]. Thence, we speculated that midazolam may modulate NSCLC cells via EGFR pathway.

Plenty of researches have justified that EGFR serves as a momentous participant in the differentiation, renewal, and growth of mammalian cells [32]. Recently, the roles of EGFR in the development of human cancer have attracted increasing attention [33]. It is widely documented that EGFR can exert its regulatory role in multiple malignancies by activating MEK/ERK signaling pathway [34–37]. For example, chaetoglobosin G hinders cell proliferation and autophagy in lung cancer via EGFR/MEK/ERK network [18]. 20(*S*)-Ginsenoside Rg3 plays an inhibitory role in lung cancer through regulating EGFR-dependent Ras/Raf/MEK/ERK pathway [38]. Therefore, to further explore the molecular mechanism underlying midazolam in NSCLC progression, we identified the potential of midazolam in EGFR/MEK/ERK pathway. Our findings showed that midazolam blunted the activation of EGFR/MEK/ERK signaling. In cell experiment, EGF stimulation antagonized midazolam-mediated EGFR inhibition and phenotypically promoted the aggressivenes of NSCLC cells. Therefore, midazolam may inhibit the development of NSCLC by inactivating EGFR/MEK/ERK pathway.

To the best of our knowledge, this research was the first to expound the association between midazolam and EGFR-mediated MEK/ERK signaling and reveal a novel molecular mechanism of midazolam in NSCLC. Mechanistically, midazolam inhibited the proliferation and migration of NSCLC cells by associating with the EGFR/MEK/ERK suppression. This may provide a novel strategy for NSCLC treatment. However, there are some limitations that remain to be improved in the future. The current data did not fully demonstrate the growth inhibition of midazolam to the EGFR signal suppression. Overexpression of EGFR rescuing midazolam-mediated phenotype experiments should be combined for verification. In addition, the potential specificity concerns such as whether the compound could have other targets in addition to EGFR, which might contribute to the observed cell growth suppression should also be studied.

## Appendix

**Table A1:** Number of voluntary activities of mice

Group	Day 7	Day 14	Day 21	Day 28
Control	110.62 ± 29.18	99.14 ± 13.29	120.10 ± 27.72	93.62 ± 13.85
Midazolam	93.62 ± 13.85	95.88 ± 10.29	98.99 ± 13.21	89.63 ± 10.85
*P*-value	0.9997	>0.9999	0.5286	0.7318

Day 7 was defined as the day on which the tumor was judged to have formed if the diameter was larger than 5.0 mm.
